# Effects of eccentric- and concentric-based plyometric programmes on strength, speed and tensiomyography parameters of female athletes

**DOI:** 10.5114/biolsport.2026.152343

**Published:** 2025-07-16

**Authors:** Nikola Prvulović, Ana Lilić, Saša Pantelić, Milan Čoh, Milica Kojadinović, Vesna Vučić, Boštjan Šimunič

**Affiliations:** 1 Group for Nutritional Biochemistry and Dietology, Institute for Medical Research, National Institute of Republic of Serbia, University of Belgrade, Serbia; 2 Faculty of sport and physical education, University of Niš, Serbia; 3 Faculty of sport, University of Ljubljana, Slovenia; 4 Center of Research Excellence in Nutrition and Metabolism, Institute for Medical Research, National Institute of Republic of Serbia, University of Belgrade, Serbia; 5 Institut for Kinesiology, Science and Research Centre of Koper, Slovenia

**Keywords:** Tensiomyography, Plyometric, Countermovement jump, Strength, Speed, Eccentric-concentric con-traction

## Abstract

The aim of the study was to examine the effects of eccentric- and concentric-based plyometric programmes on the strength, speed, and tensiomyography (TMG) parameters of female athletes. The study included twenty junior female participants from three different sports equally divided into two experimental groups of n = 10. Two plyometric programmes with contrasting designs were conducted for a period of six weeks, with sessions held twice per week. The first plyometric programme (ECC-CON-G) was based on exercises with eccentric, and the second (CONC-CON-G) with concentric contractions. TMG was used to evaluate neuromuscular performances of six muscles of both legs – vastus lateralis (VL), vastus medialis (VM), biceps femoris (BF), semitendinosus (SM), gastrocnemius lateralis (GL), and gastrocnemius medialis (GM)) – and two strength and speed tests: countermovement jump (CMJ), and sprint test at 10 m and 20 m. The results show that both groups had significant effects between pre-post measurements in CMJ (Diff, ECC-CON-G = 9.02%, and CONC-CON-G = 5.59%, p < 0.05), at 10 m (Diff, ECC-CON-G = 9.23%, and CONC-CON-G = 9.35%, p < 0.001), and 20 m (Diff, ECC-CON-G = 6.16%, and CONC-CON-G = 5.35%, p < 0.001), and TMG parameters in ECC-CON-G (all 6 left leg muscles, and right leg-VL, BF, GL, GM, p < 0.05), in CONC-CON-G (left leg-BF, SM, GL, GM, and right leg-VL, BF, GL, GM, p < 0.05). There were significantly better effects in ECC-CON-G compared to CONC-CON-G for CMJ height and time, for only time in 20 m sprint, and TMG parameters for left leg VL and VM, and right leg BF and GM. A plyometric programme based on exercises with eccentric contractions proved more beneficial for strength, speed, and TMG parameters in young female athletes compared to a programme based on concentric contractions.

## INTRODUCTION

Plyometric programmes are known for their benefits in developing strength and speed and are inevitable in all explosively demanding sports for both genders [1]. The key to their success lies in the third phase of the plyometric action, known as the coupling time, which occurs between the end of the eccentric phase and the start of the concentric phase [1]. The most compelling evidence of their effects comes from meta-analyses, which highlight the optimal duration, frequency, and intensity of programmes lasting 6 to 10 weeks (positive effects have also been observed with shorter programmes) [2–4]. These programmes typically involve two or more high-intensity training sessions per week, each lasting 45 to 60 minutes, with 600 to 900 repetitions of various plyometric and other jump exercises [1, 5]. The effects are measured and tracked using various speed [6] and strength tests, as well as vertical jumps, with the countermovement jump (CMJ) being the primary assessment [7].

In addition to these tests, the assessment of athletes’ muscle capacitive properties using tensiomyography (TMG) [8–13] is becoming increasingly common in both scientific research and training programmes. TMG-derived contraction time (Tc) and displacement amplitude (Dm) were analysed after 9-week plyometric training regimes in young [11] and old individuals [12]. Both studies reported Tc and Dm decreases, but they were not consistent in all muscles. As Tc was previously correlated with muscle fibre type composition [14], it was assumed that plyometric training is beneficial for increasing the proportion of type II muscle fibres. Dm was previously correlated with muscle atrophy after bed rest and hypertrophy after the rehabilitation protocols [15, 16]. As Dm adaptations preceded structural adaptations, it is assumed that Dm reflects muscle stiffness changes [15, 16], which are regularly affected by plyometric training [17]. On the other hand, TMG-derived delay time (Td), sustain time (Ts) and half-relaxation time (Tr) are rarely investigated. However, Td (an electromechanical delay) and Tr also correlated with muscle composition [14].

Training programmes, including plyometrics, enhance strength and speed by repeating exercises of varying intensity, thereby preparing the central nervous system (CNS) for upcoming demands [1, 18]. One of the main roles of the CNS is to protect and adapt the body to external loads or exercises that require the generation of large forces [1]. The effects of exercise are visible in the muscles as hypertrophy [19], including both sarcoplasmic and sarcomeric hypertrophy, increased energy storage in the CNS, and greater muscle tone [18].

Numerous studies have highlighted the positive effects of plyometric training in various athletes, but few have focused on younger, junior-age athletes [5, 20]. Additionally, the number of studies involving younger female athletes is limited [5, 20].

Almost every plyometric programme includes some form of exercise based on concentric contractions (CONC-CON), such as the squat jump (SJ) [3, 21, 22], while exercises based on eccentric contractions (ECC-CON) are quite rare [23, 24, 25]. Verkhoshansky referred to this as pseudo-plyometrics, or depth landings from different heights (DL), which are unique in producing maximum involuntary contractions triggered by the impact of the body. These contractions cannot be generated by weight training or any other similar methods of resistance training or jumps [1]. The growing interest in eccentric-based exercises is evident in recent literature, where flywheels are used for CONC-CON followed by ECC-CON, or in the concept of “eccentric overload” [10].

Every human movement or exercise is generated by muscle contractions, which can be classified as concentric, eccentric, isometric, or isokinetic [1]. Each type of muscle contraction has its own specific properties that impact the neuromuscular system when exercising or “stimulating” the muscles. It leads to adaptations in the neuromuscular system, preparing it for future exercises [1, 26, 27]. Understanding the differences between exercises, specifically the effects of muscle contractions, contributes to enhancing the training process and the development of motor skills and strength, and ultimately improves sports performance both directly and indirectly.

The effects of either solely concentric or mixed ECC-CON and CONC-CON exercises have been analysed previously [19]. The greatest progress in strength and speed was observed in the mixed programme, followed by the programme with CONC-CON followed by ECC-CON. Both of these programmes resulted in significant improvements compared to the programme with exclusively CONC-CON. Exercises with ECC-CON followed by CONC-CON lead to the greatest improvements in hypertrophy, nerve activation, and strength of the exercised muscles [26]. The vastus lateralis shows greater activity after eccentric training compared to concentric training [26]. Pseudo-plyometrics, as mentioned, have been scarcely researched, particularly regarding their effect on developing explosive power [1, 23]. DL have been more commonly used in experiments to analyse the development of stability in the knee and ankle joints of gymnasts [23, 25, 29]. Although the positive effects have been proven, the experiments were discontinued due to the risk of injury [1, 23, 28]. DL were performed from extreme heights (3.2 m) and in combination with other exercises, which further increased the strain on the neuromuscular system of the subjects [23]. As a result, there has been less experimentation with pseudo-plyometric exercises. Therefore, our experiment took advantage of this fact and aimed to optimize the use of this specific type of exercise in training cycles and programmes, but with smaller heights.

The possibility for combining exercises with ECC-CON and oppositely conceived plyometric exercise programmes, with TMG measurements, can provide new knowledge and possibly improve the existing state of strength, speed, and contractile ability of the muscles. Therefore, the aim of the study was to determine the effects of eccentric- and concentric-based plyometric programmes on the strength, speed, and TMG parameters of female athletes.

## MATERIALS AND METHODS

### Experimental design

An experimental longitudinal controlled study was conducted with female athletes participating in three distinct sports – athletics (sprinting disciplines), basketball, and volleyball – all competing at the national league level, divided into two experimental groups, ECC-CON-G (eccentric plyometric intervention) and CONC-CON-G (concentric plyometric intervention). The study analysed improvements after a 6-week intervention in strength (using the CMJ test), speed (through sprint tests), and neuromuscular performance (measured using TMG) on six muscles of the lower extremities.

### Participants

Initially, 24 female athletes aged 16 to 18 years volunteered to participate in the study. Eight participants were from sports where explosive strength is a key motor skill for sporting success, including basketball, volleyball, and athletics (sprint disciplines). All participants had been active in their respective sports for at least 3 years and injury-free for at least one year prior to the study. After the study protocol received approval from the Ethics Committee of the Faculty of Sport and Physical Education, University of Nis (number: 04-227/2), and prior to familiarization and data collection sessions, the parents and coaches of minor participants were fully informed about the goals, course, participation and possible side effects of the research and signed informed written consent before the start of the study. Adult participants were also fully informed verbally and signed informed written consent before the study. The study protocol was conducted according to the Declaration of Helsinki [29]. The participants were divided into two groups of 12, each following a different plyometric programme. The equality of the groups was assessed based on the mean values of their morphological characteristics (BH – body height, BM – body mass). The first plyometric programme was based on exercises with ECC-CON, and the second on CONC-CON. The final sample included 20 participants (ECC-CON-G, n = 10, age: 17.0 ± .94 years, height: 174.9 ± 4.75 cm, mass: 65.5 ± 6.68 kg; CONC-CON-G, n = 10, age: 16.9 ± 1.1 years, height: 171.9 ± 8.36 cm, mass: 65.8 ± 9.6 kg) who successfully completed the experimental protocol.

### Procedure

Before the initial measurements, participants were instructed to refrain from physical activity for 2 days. All participants were measured at the same time, both immediately before and two days after the applied programmes, during the initial and final measurements. All measurements (pre- and post-) were conducted 10 minutes after the warm-up, using the same measuring devices and performed by the same researchers. Prior to the measurements, a 20-minute warm-up was completed, including neuromuscular activation exercises and lower-body dynamic stretches to prepare for the upcoming tests.

### Anthropometric measurements

All anthropometric measurements were conducted according to international standards for anthropometric assessment [30] and were performed before any exercise programme. To ensure precise testing, participants were asked to be barefoot and wear light clothing. Anthropometric measurements were obtained using a mobile stadiometer (SECA 217, Hamburg, Germany) and an electronic scale (SECA 803, Hamburg, Germany). Body height was measured with the accuracy of ± 0.1 cm, and body mass with the accuracy of ± 0.1 kg.

### Countermovement jump (CMJ)

The CMJ without an arm swing was used to assess lower-body muscular power [7]. Participants were instructed to jump as high as possible and land in a position similar to the take-off position on the force plate (with straight knees and plantar-flexed ankles). The subject’s maximum CMJ height and kinetic parameters were measured using a force plate (Kistler 9286A) with a sampling rate of 1000 Hz. Participants practised the CMJ task twice, followed by a 2-minute rest period before performing 3 maximal jumps, which were recorded and used for analysis.

### Sprint

Ten and 20 m sprint tests were used to assess speed [6]. The subject speed was measured using three pairs of photocells (Witty · Gate, Microgate, Italy) placed at the start, 10 m, and 20 m. A standing static start (front foot placed 0.5 m behind the start line) and a photocell trigger were used to ensure enough space for the subject’s head to be positioned just behind the starting beam of the photocells [6]. A total of three maximum attempts at 20 m, with the passing time recorded at 10 m, were made, and the fastest time was calculated.

### TMG measurement

TMG was used to assess skeletal muscle contractile properties in the vastus lateralis (VL), vastus medialis (VM), biceps femoris (BF), semitendinosus (SM), gastrocnemius lateralis (GL), and gastrocnemius medialis (GM) of both legs. All measurements were performed isometrically in relaxed predefined positions: for VL and VM in supine position with the knee angle set at 30° flexion (where 0° represents the extended joint); for BF and SM in prone position with the knee angle set at 5° flexion; for GL and GM in prone position with the ankle in neutral position [14]. Foam pads were used to support the joints. The sensor was positioned at the thickest part of each muscle belly, perpendicular to the tangential plane on the skin above the measuring point. A 1 ms rectangular (twitch) impulse was applied through selfadhesive stimulation electrodes (5 × 5 cm Compex Medical AS, Ecublens, Switzerland) that were positioned 5 cm distally (cathode) and 5 cm proximally (anode) to the measuring point, following the arrangement of the fibres [9]. An electrical stimulator was used (TMG-S2, TMG-BMC, Slovenia), and a digital high-precision displacement sensor (digital–optical comparator, TMG-BMC Ltd, Slovenia) that was pressed by a spring (.2 N/cm^2^) on the muscle belly during the measurement, to assure a high signal-to-noise ratio and high reliability [31]. Initially, the electrical current amplitude was set just above the threshold (~20 mA) and was then gradually increased until the TMG amplitude and Dm readings stabilized at maximal values. When necessary, the measuring point and electrode positions were adjusted to obtain maximum Dm of the muscle belly. From two maximal twitch responses, the muscle contractile properties were calculated, and an average was used for further analysis [32]. To avoid fatigue or potentiation effects, a 15 s resting period was allowed between electrical stimuli [13]. Both TMG (pre-test and post-test) tests were conducted in the morning by the same experienced specialist. Altogether, five TMG-derived contractile parameters were calculated: Dm, delay time Td as the time from the electrical pulse to the time that the contraction reached 10% of the Dm, sustain time Ts as the time when the contraction was above 50%, half-relaxation time Tr as the time for contraction to decrease from 90% to 50% Dm, and contraction time Tc as the time for contraction to increase from 10% to 90% of Dm [12]. The high reliability of TMG-derived parameters (Td, Tc, and Dm) is emphasized, while Tr is moderately reliable [33].

### Experimental training interventions

Before the start of the experimental plyometric programmes, one week was dedicated to familiarizing the participants with the measuring instruments and testing rooms, as well as providing detailed explanations of the procedure and objectives of the experiment. This was followed by the initial measurements for all assigned tests and the implementation of the planned plyometric programmes (see Table 1).

**TABLE 1:** Characteristics of the plyometric programs

Characteristics of the program	Experimental plyometric program based on ECC-CON-G	Experimental plyometric program based on CONC-CON-G
Duration	6 weeks	6 weeks
Frequency	2 per week	2 per week
Duration of training	45–60 min	45–60 min
Breaks between jumps	1–5 seconds	1–5 seconds
Breaks between sets	60–120 seconds	60–120 seconds
Breaks between exercises	120–240 seconds	120–240 seconds
Intensity	High	High
Ratio between all jumps, and DL or SJ	30–40% to 70–60%.	30–40% to 70–60%.
Structure	Three-part	Three-part
Number of exercises	12 (4 for warm up)	12 (4 for warm up)
Type of exercise	Horizontal standing long jump, standing triple jump, horizontal vertical double jump from a standing position from box (30 cm), Drop jump (30 cm) with double rebound, sprint (10 m and 20 m), Depth landings (from different heights 60–100 cm).	Horizontal standing long jump, standing triple jump, horizontal vertical double jump from a standing position from box (30 cm), Drop jump (30 cm) with double rebound, sprint (10 m and 20 m), Squat jump (jump on the different height boxes 30–60 cm).
Note	The structure of the training sessions was based on previous research and guidelines, intensity was based on the length of breaks between jumps, exercises and sets in study [5], and the type of exercise [1]. The total number of jumps in a set as well as the number of sets decreased as landing height or SJ increased during the program [1, 5, 23].

Legend: ECC-CON-G – Eccentric plyometric intervention group; CONC-CON-G – Concentric plyometric intervention group.

### Statistical analysis

The data and all statistical analyses were conducted using the IBM SPSS Statistics for Windows software (Version 28.1; IBM Corp., Armonk, NY). All obtained data are represented by descriptive statistics parameters (central and dispersive parameters: arithmetic mean (mean), standard deviation (std. deviation). Normality was assessed using the Kolmogorov-Smirnov test. The homogeneity of variances was confirmed using Levene’s test. Student’s independent t-test was used to determine differences between groups at the initial measurements, and two-way repeated measures ANOVA was used to determine the effects of different plyometric programmes over time (pre- and post-test). The degree of the effect for dependent variables was determined using partial eta squared (ƞp^2^). Partial eta squared values of .01, .06, and .14 were considered small, moderate, and large effects, respectively [34]. Due to the dual approach for checking and analysing the results, a one-way analysis of covariance (ANCOVA) (using baseline values as covariates) was performed to assess the effects and differences between the plyometric programmes [35]. Statistical significance was set at p < 0.05.

## RESULTS

There were no significant differences between the groups in the initial measurements of anthropometric parameters (p > 0.05).

Table 2 displays the differences between the initial and final measurements, showing a large significant effect in the ECC-CON-G for three measured CMJ parameters (Height, Rel F and Rel I, p < 0.05), and in the CONC-CON-G for two CMJ parameters (Height and Rel I, p < 0.05). Both groups showed significant results with a large effect for time in the 10 m and 20 m sprint tests, respectively (p < 0.001).

**TABLE 2:** Initial and final results, and differences between initial and final measurement for both groups on the CMJ test and 10 m and 20 m sprint tests.

Tests	Variables	G	Pre-test (M+Sd)	Post-test (M+Sd)	Diff (%) Pre-Post	ηp^2^
Anthropometric	Age (years)	ECC-CON-G CON-CON-G	17.0 ± 0.9 16.9 ± 1.1	N/A	N/A	N/A

	Height (cm)	ECC-CON-G CON-CON-G	174.9 ± 4.7 171.9 ± 8.4			

	Mass (kg)	ECC-CON-G CON-CON-G	65.5 ± 6.7 65.8 ± 9.6			

CMJ	Height (cm)	ECC-CON-G CON-CON-G	37.9 ± 4.7 37.0 ± 4.1	41.3 ± 3.5 39.1 ± 3.7	3.4 (9.0) ^***^ 2.1 (5.6)^*^	**0.79** **0.53**

	Time (s)	ECC-CON-G CON-CON-G	0.8 ± 0.1 0.8 ± 0.2	0.88 ± 0.13 0.76 ± 0.13^#^	0.08 (10.0) -0.04 (5.0)	0.24 0.09

	Rel F (F · Ns)	ECC-CON-G CON-CON-G	227.3 ± 19.8 226.1 ± 27.2	244.3 ± 19.8 233.0 ± 29.6	17.0 (7.5)^*^ 6.9 (3.0)	**0.59** 0.24

	Rel I (N · m/s)	ECC-CON-G CON-CON-G	147.2 ± 21.0 143.6 ± 21.0	156.3 ± 19.0 150.3 ± 20.1	9.1 (6.2)^*^ 6.8 (4.7)^*^	**0.58** **0.60**

Sprint	10 m (s)	ECC-CON-G CON-CON-G	1.95 ± 0.1 2.03 ± 0.1	1.76 ± 0.1 1.84 ± 0.1^#^	-0.18 (9.2)^***^ -0.19 (9.3)^***^	**0.77** **0.80**

	20 m (s)	ECC-CON-G CON-CON-G	3.41 ± 0.1 3.55 ± 0.2	3.19 ± 0.1 3.36 ± 0.1^#^	-0.21 (6.2)^***^ -0.19 (5.3)^***^	**0.83** **0.78**

Legend: G – Group; M – mean; Sd – standard deviation; pre-test; initial measurement; Post-test – final measurement; Diff – difference between the initial and final measurements; ηp^2^ – partial eta square; Height – jump height; Rel F – total relative force; Time – CMJ jump time; Rel I – total relative impulse; ECC-CON-G – Eccentric plyometric intervention group; CONC-CON-G – Concentric plyometric intervention group; $ – statistically significant result at the pre-test, p < 0.05;

# – statistically significant result at the post-test, p < 0.05;

* – statistically significant result between the pre- and post-test; p < 0.05;

*** – statistically significant result, p < 0.001; N/A – not applicable.

**FIG. 1:**
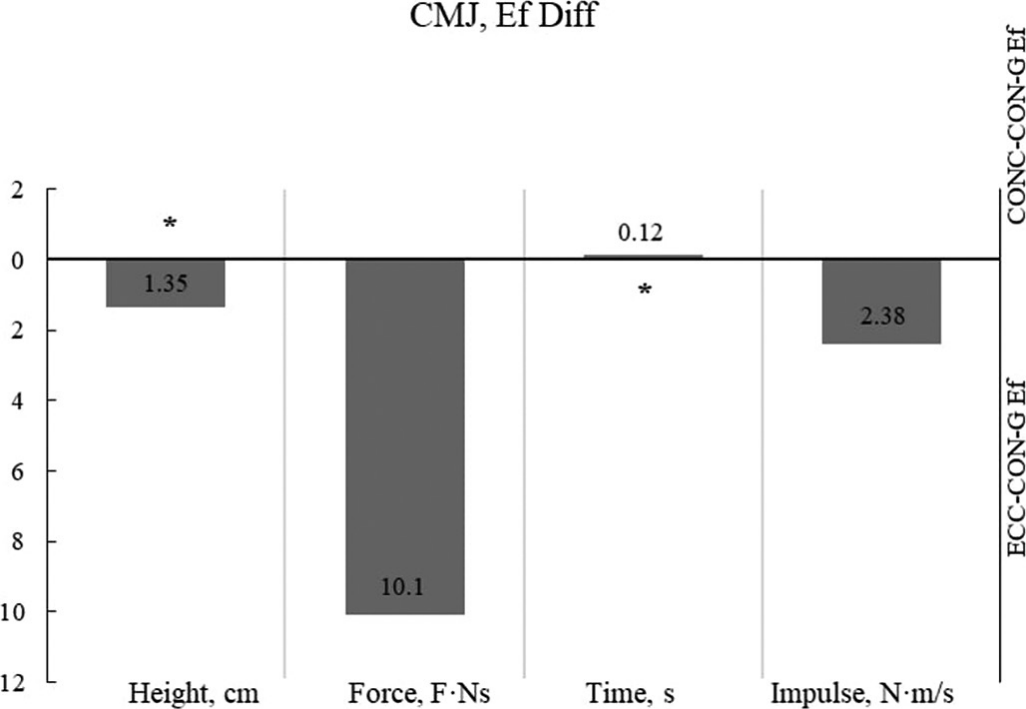
Differences in the effects of the plyometric programs on CMJ kinetic parameters Legend: * – statistically significant result with p< 0.05; ECC-CON-G – Eccentric plyometric intervention group; CONC-CON-G – Concentric plyometric intervention group. Note: Ef Diff were calculated by subtracting the results of the mean values ECC-CON-G from CONC-CON-G. All results are presented as absolute values on both sides of the Y-axis: below the X-axis as the ECC-CONC-G effect side, and above the x-axis as the CON-CONC-G effect side.

Figure 1 displays the effects on the CMJ kinetic parameters, with a significant large effect observed for two of the four measured variables: Height and Time.

**FIG. 2:**
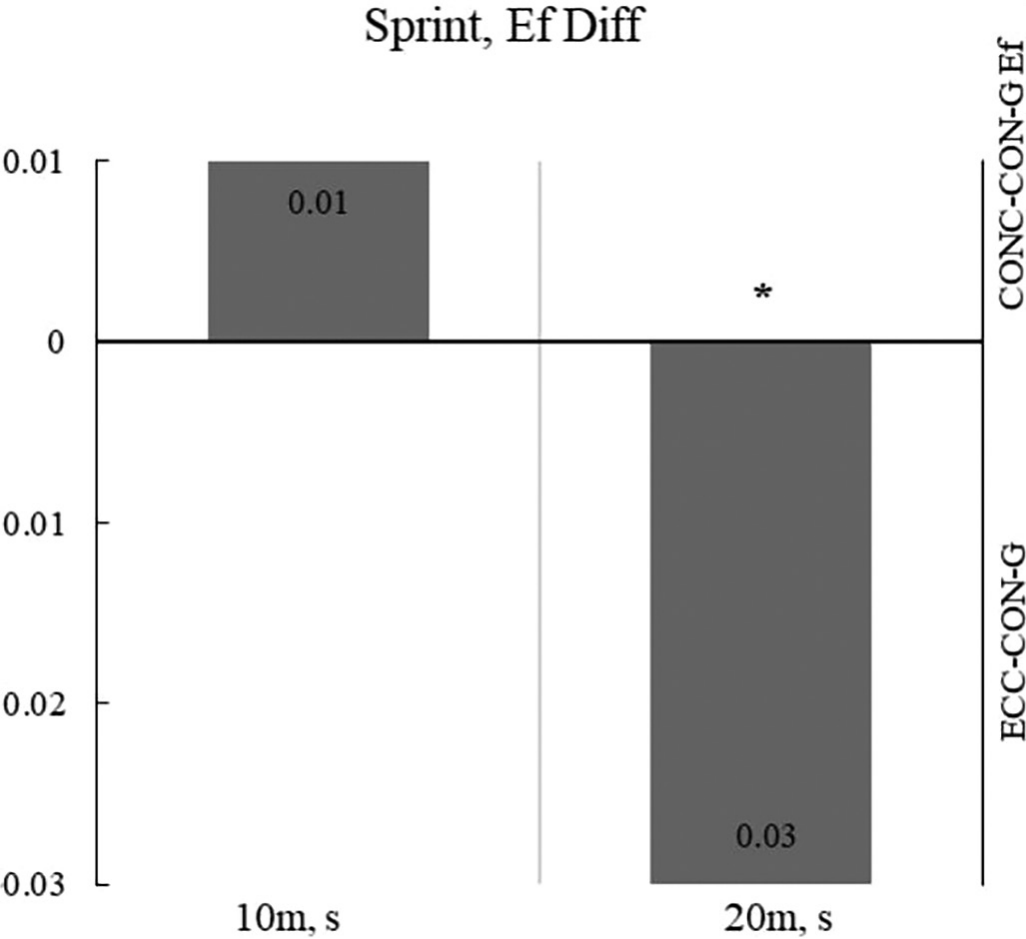
Differences in the effects of the plyometric programs on the 10 m and 20 m spring tests Legend: * – statistically significant result with p< 0.05; ECC-CON-G – Eccentric plyometric intervention group; CONC-CON-G – Concentric plyometric intervention group. Note: Ef Diff were calculated by subtracting the results of the mean values ECC-CON-G from CONC-CON-G. All results are presented as absolute values on both sides of the Y-axis: below the X-axis as the ECC-CONC-G effect side, and above the x-axis as the CON-CONC-G effect side.

Figure 2 displays the effects on the 10 m and 20 m sprint tests, with a significant large effect observed only for the 20 m sprint test.

Table 3 displays the differences between the initial and final measurements, showing a significant effect for TMG parameters in the ECC-CON-G. This was observed in both extensor muscles of the left lower leg: VL (Ts, Tr, and Dm, p < 0.05) and VM (Tc and Ts, p < 0.05). In the right leg, significant effects were found only for VL (Ts, Tr, and Dm, p < 0.05). Significant results in the CONC-CON-G were only observed for the muscles of the right leg, specifically VL (Ts, Tr, and Dm, p < 0.05) and VM (Ts, p < 0.05).

**TABLE 3:** TMG parameters of the lower leg extensor muscles at the initial and f‍inal measurements, and differences between the initial and final measurement for both groups.

TMG	Leg	G	m.Vastus lateralis			

			Pre-test (M+Sd)	Post-test (M+Sd)	Diff (%) Pre-Post	ηp^2^
Tc (ms)	Left	ECC-CON-G CON-CON-G	20.4 ± 3.7 24.0 ± 5.3	22.3 ± 1.2 21.6 ± 2.4	1.9 (9.3) -2.4 (10.0)	0.30 0.26

	Right	ECC-CON-G CON-CON-G	19.3 ± 5.5 22.2 ± 4.8	22.5 ± 1.8 22.8 ± 2.3	3.2 (16.4) 0.5 (2.4)	0.25 0.03

Ts (ms)	Left	ECC-CON-G CON-CON-G	64.3 ± 47.3 87.1 ± 44.1	116.5 ± 41.9 109.4 ± 54.4	52.3 (81.3)^*^ 22.4 (34.3)	**0.59** 0.33

	Right	ECC-CON-G CON-CON-G	48.2 ± 42.4 80.6 ± 48.6	115.4 ± 19.9 113.3 ± 40.2	67.3 (139.7)^*^ 32.7 (40.6)^*^	**0.72** **0.41**

Tr (ms)	Left	ECC-CON-G CON-CON-G	35.5 ± 37.2 50.4 ± 31.4	86.8 ± 39.6 67.7 ± 48.7	51.3 (144.4)^*^ 17.3 (25.7)	**0.59** 0.20

	Right	ECC-CON-G CON-CON-G	21.5 ± 26.6 43.2 ± 32.6	86.3 ± 19.4 68.9 ± 35.9	64.8 (301.0)^***^ 25.6 (59.4)^*^	**0.81** **0.38**

Td (ms)	Left	ECC-CON-G CON-CON-G	21.1 ± 1.7 21.9 ± 2.0	22.3 ± 0.7 21.2 ± 1.3#	1.1 (5.3) -0.8 (3.5)	0.30 0.22

	Right	ECC-CON-G CON-CON-G	20.3 ± 2.0 24.6 ± 10.5	21.5 ± .6 21.1 ± 1.1	1.2 (5.8) -3.8 (14.1)	0.21 0.10

Dm (mm)	Left	ECC-CON-G CON-CON-G	2.8 ± 1.3 3.9 ± 1.0$	4.5 ± 1.2 4.3 ± 1.2	1.7 (60.4)^*^ 0.4 (10.3)	**0.61** 0.11

	Right	ECC-CON-G CON-CON-G	2.2 ± 1.3 2.9 ± 0.6	4.3 ± 1.1 4.5 ± 0.8	2.2 (101.4)^*^ 1.6 (54.1)^***^	**0.60** **0.82**

**m.Vastus medialis**						

Tc (ms)	Left	ECC-CON-G CON-CON-G	23.3 ± 1.2 22.3 ± 1.6	24.9 ± 1.8 22.4 ± 2.5#	1.6 (6.7)^*^ 0.1 (0.4)	**0.62** 0.01

	Right	ECC-CON-G CON-CON-G	22.9 ± 3.1 26.6 ± 9.5	23.6 ± 2.4 23.2 ± 2.9	0.6 (2.7) -3.4 (12.7)	0.06 0.20
Ts (ms)	Left	ECC-CON-G CON-CON-G	168.8 ± 28.9 177.6 ± 32.4	196.5 ± 44.4 182.7 ± 38.4	27.7 (16.4)^*^ 5.1 (2.8)	**0.65** 0.04

	Right	ECC-CON-G CON-CON-G	183.2 ± 26.4 171.5 ± 16.4	238.4 ± 124.9 193.0 ± 12.8	55.2 (30.1) 21.5 (12.5)^*^	0.24 **0.56**

Tr (ms)	Left	ECC-CON-G CON-CON-G	58.7 ± 39.9 39.8 ± 17.6	68.0 ± 45.7 52.0 ± 37.0	9.2 (15.7) 12.2 (30.6)	0.04 0.09

	Right	ECC-CON-G CON-CON-G	54.4 ± 33.6 71.6 ± 55.3	51.3 ± 17.4 53.3 ± 44.9	-3.1 (5.8) -18.3 (25.5)	0.01 0.08

Td (ms)	Left	ECC-CON-G CON-CON-G	21.1 ± 1.9 20.9 ± 1.5	21.5 ± 1.9 21.2 ± 0.6	0.4 (1.8) 0.3 (1.6)	0.10 0.04

	Right	ECC-CON-G CON-CON-G	22.4 ± 1.6 22.8 ± 1.5	21.8 ± 1.5 21.8 ± 2.1	-0.6 (2.8) -0.9 (4.1)	0.26 0.28

Dm (mm)	Left	ECC-CON-G CON-CON-G	4.9 ± 1.7 5.1 ± 1.6	5.0 ± 2.0 5.3 ± 1.5	0.1 (2.6) 0.2 (3.7)	0.01 0.01

	Right	ECC-CON-G CON-CON-G	5.7 ± 1.8 6.1 ± 1.5	5.4 ± 1.4 5.9 ± 1.4	-0.3 (6.1) -0.2 (4.1)	0.08 0.02

Legend: M – mean; Sd – standard deviation; pre-test; initial measurement; Post-test – final measurement; Diff – difference between the initial and final measurements; ηp^2^ – partial eta square; Tc – contraction time; Ts – sustain time; Tr – relaxation time; Td – delay time; Dm – maximal displacement; ECC-CON-G – Eccentric plyometric intervention group; CONC-CON-G – Concentric plyometric intervention group;

* – statistically significant result, p < 0.05;

** – statistically significant result, p < 0.001.

Table 4 displays the differences between the initial and final measurements, showing a large significant effect for TMG parameters in both calf flexor muscles. Both groups exhibited similar results for the left lower leg, BF (ECC-CON-G-Tr and Dm, CONC-CON-G-Dm, p < 0.05) and SM (ECC-CON-G-Ts, and CONC-CON-G-Tc, p < 0.05). In the right leg, significant effects were observed only for BF (ECC-CON-G-Td and Dm, and CONC-CON-G-Tc, Td, and Dm, p < 0.05).

**TABLE 4:** TMG parameters of the flexor’s muscles of the lower leg at the initial and final measurement, and differences between the initial and final measurement for both groups.

TMG	Leg	G	m.Biceps femoris			

			Pre-test (M+Sd)	Post-test (M+Sd)	Diff (%) Pre-Post	ηp^2^
Tc (ms)	Left	ECC-CON-G CON-CON-G	40.1 ± 19.6 33.2 ± 15.2	44.3 ± 12.4 42.3 ± 16.7	4.2 (10.4) 9.0 (27.2)	0.04 0.27

	Right	ECC-CON-G CON-CON-G	32.9 ± 13.3 27.3 ± 11.9	35.5 ± 7.7 47.1 ± 15.0#	2.6 (8.0) 19.8 (72.4)^*^	0.03 **0.64**

Ts (ms)	Left	ECC-CON-G CON-CON-G	142.0 ± 65.5 199.0 ± 62.5	183.3 ± 21.7 183.2 ± 17.1	41.3 (29.1) -15.8 (7.9)	0.30 0.08

	Right	ECC-CON-G CON-CON-G	222.0 ± 80.0 122.3 ± 75.0$	184.1 ± 21.7 175.3 ± 18.9	-37.9 (17.1) 53.0 (43.3)	0.23 0.30

Tr (ms)	Left	ECC-CON-G CON-CON-G	45.2 ± 20.1 51.6 ± 35.7	79.5 ± 32.7 65.2 ± 25.9	34.3 (75.9)^*^ 13.5 (26.2)	**0.54** 0.20

	Right	ECC-CON-G CON-CON-G	74.9 ± 50.2 48.8 ± 41.5	59.6 ± 16.6 70.2 ± 23.2	-15.3 (20.4) 21.4 (43.8)	0.08 0.19

Td (ms)	Left	ECC-CON-G CON-CON-G	25.1 ± 3.8 23.5 ± 4.0	27.1 ± 2.3 25.5 ± 3.6	2.0 (8.1) 2.0 (8.6)	0.31 0.34

	Right	ECC-CON-G CON-CON-G	23.4 ± 3.1 21.9 ± 3.0	26.3 ± 1.7 26.8 ± 2.9	2.9 (12.4)^*^ 4.6 (21.2)^*^	**0.61** **0.71**

Dm (mm)	Left	ECC-CON-G CON-CON-G	4.5 ± 2.4 3.7 ± 1.8	7.5 ± 1.7 5.7 ± 2.0#	3.0 (67.2)^*^ 2.0 (53.1)^*^	**0.60** **0.55**

	Right	ECC-CON-G CON-CON-G	3.0 ± 1.1 2.2 ± 1.8	6.6 ± 2.2 5.6 ± 1.1	3.6 (118.2)^*^ 3.4 (150.4)^***^	**0.69** **0.82**

**m.Semitendinosus**						

Tc (ms)	Left	ECC-CON-G CON-CON-G	46.4 ± 8.4 45.2 ± 8.0	37.9 ± 11.3 35.7 ± 10.0	-8.5 (18.3) -9.4 (20.9)^*^	0.22 **0.46**

	Right	ECC-CON-G CON-CON-G	43.2 ± 7.7 40.7 ± 12.6	37.4 ± 7.3 38.3 ± 11.7	-5.8 (13.5) -2.7 (5.8)	0.19 0.10

Ts (ms)	Left	ECC-CON-G CON-CON-G	151.4 ± 30.9 159.2 ± 27.4	181.4 ± 33.6 167.5 ± 32.0	30.0 (19.8)^*^ 8.2 (5.2)	**0.38** 0.07

	Right	ECC-CON-G CON-CON-G	179.8 ± 70.3 158.5 ± 25.5	178.2 ± 35.1 170.6 ± 13.6	-1.6 (.9) 12.1 (7.6)	0.01 0.31

Tr (ms)	Left	ECC-CON-G CON-CON-G	78.4 ± 27.6 72.6 ± 28.3	87.3 ± 49.5 78.6 ± 30.9	8.8 (11.2) 6.0 (8.3)	0.05 0.03

	Right	ECC-CON-G CON-CON-G	77.5 ± 39.8 73.7 ± 31.0	97.1 ± 50.1 91.9 ± 30.1	19.6 (25.3) 18.2 (24.7)	0.34 0.31

Td (ms)	Left	ECC-CON-G CON-CON-G	25.8 ± 2.1 27.2 ± 2.2	25.1 ± 3.3 27.1 ± 7.8	-0.7 (2.7) -0.1 (.37)	0.04 0.01

	Right	ECC-CON-G CON-CON-G	27.0 ± 5.9 25.1 ± 3.6	24.2 ± 2.8 25.4 ± 2.6	-2.7 (10.2) 0.3 (1.2)	0.21 0.01

Dm (mm)	Left	ECC-CON-G CON-CON-G	6.0 ± 1.9 6.0 ± 2.2	5.8 ± 2.3 5.7 ± 2.9	-0.2 (3.3) -0.2 (4.0)	0.01 0.01

	Right	ECC-CON-G CON-CON-G	5.7 ± 2.0 5.9 ± 2.2	5.7 ± 1.9 6.0 ± 1.9	-0.1 (1.4) 0.1 (1.3)	0.01 0.01

Legend: M – mean; Sd – standard deviation; pre-test; initial measurement; Post-test – final measurement; Diff – difference between the initial and final measurements; ηp^2^ – partial eta square; Tc – contraction time; Ts – sustain time; Tr – relaxation time; Td – delay time; Dm – maximal displacement; ECC-CON-G – Eccentric plyometric intervention group; CONC-CON-G – Concentric plyometric intervention group;

* – statistically significant result, p < 0.05;

** – statistically significant result, p < 0.001.

Table 5 displays the differences between the initial and final measurements, showing a large significant effect for TMG parameters in both groups. Significant effects were observed for both the left GL (ECC-CON-G-Ts and Td, and CONC-CON-G-Td) and the right foot extensor muscles, including GL (ECC-CON-G and CONC-CON-G-Ts, Td, and Dm, p < 0.05, respectively). In the left leg for GM, the ECC-CON-G showed significant effects for Tc, Tr, Td, and Dm, while the CONC-CON-G showed effects for Tc, Td, and Dm. In the right leg for GM, the ECC-CON-G showed significant effects for Tc, Tr, Td, and Dm, while the CONC-CON-G showed effects for Td and Dm (p < 0.05).

**TABLE 5:** TMG parameters of the extensor’s muscles of the foot at the initial and final measurement, and the differences between the initial and final measurement for both groups.

TMG	Leg	G	m.Gastrocnemius lateralis			

			Pre-test (M+Sd)	Post-test (M+Sd)	Diff (%) Pre-Post	ηp^2^

Tc (ms)	Left	ECC-CON-G CON-CON-G	27.5 ± 16.0 22.5 ± 8.4	28.0 ± 23.6 18.9 ± 4.2	0.5 (1.8) -3.7 (16.2)	0.01 0.17

	Right	ECC-CON-G CON-CON-G	28.8 ± 17.1 27.8 ± 12.7	23.8 ± 13.3 21.3 ± 13.6	-5 (17.4) -6.4 (23.1)	0.09 0.11

Ts (ms)	Left	ECC-CON-G CON-CON-G	191.9 ± 35.8 173.0 ± 55.1	220.2 ± 31.5 210.3 ± 23.4	28.3 (14.8)^*^ 37.3 (21.6)	**0.50** 0.33

	Right	ECC-CON-G CON-CON-G	183.3 ± 30.8 194.4 ± 20.5	218.2 ± 38.8 220.42 ± 31.1	34.8 (19.0)^*^ 26.0 (13.4)^*^	**0.56** **0.45**

Tr (ms)	Left	ECC-CON-G CON-CON-G	54.7 ± 50.8 57.7 ± 52.2	30.8 ± 22.5 22.5 ± 10.8	-24.0 (43.8) -35.2 (61.0)	0.16 0.32

	Right	ECC-CON-G CON-CON-G	43.6 ± 21.6 39.0 ± 15.4	35.5 ± 17.5 32.4 ± 24.8	-8.1 (18.6) -6.6 (17.0)	0.25 0.07

Td (ms)	Left	ECC-CON-G CON-CON-G	21.0 ± 3.3 20.6 ± 1.0	18.8 ± 2.5 18.5 ± 1.1	-2.2 (10.3)^*^ -2.1 (10.1)^*^	**0.49** **0.71**

	Right	ECC-CON-G CON-CON-G	21.4 ± 2.1 21.4 ± 1.5	18.3 ± 1.8 18.7 ± 1.4	-3.0 (14.2)^*^ -2.6 (12.3)^*^	**0.65** **0.65**

Dm (mm)	Left	ECC-CON-G CON-CON-G	2.6 ± 1.4 2.4 ± 1.6	2.1 ± 1.1 2.2 ± 1.3	-0.5 (19.0) -0.1 (6.2)	0.33 0.02

	Right	ECC-CON-G CON-CON-G	3.4 ± 1.8 3.1 ± 1.0	2.1 ± .7 .9 ± 1.4	-1.3 (39.2)^*^ -1.2 (39.7)^*^	**0.52** **0.64**

**m.Gastrocnemius medialis**						

Tc (ms)	Left	ECC-CON-G CON-CON-G	16.3 ± 3.7 16.7 ± 3.7	20.3 ± 1.8 20.8 ± 2.8	4.0 (24.4)^*^ 4.0 (24.0)^*^	0**.52** 0**.57**

	Right	ECC-CON-G CON-CON-G	15.3 ± 3.1 17.8 ± 4.5	21.1 ± 1.3 20.2 ± 43	5.7 (37.3)^***^ 2.3 (13.2)	0**.81** 0.17

Ts (ms)	Left	ECC-CON-G CON-CON-G	181.0 ± 93.7 107.4 ± 101.8	231.7 ± 26.3 164.2 ± 90.6	50.8 (28.1) 56.8 (52.9)	0.25 0.34

	Right	ECC-CON-G CON-CON-G	168.9 ± 107.7 208.7 ± 73.2	234.6 ± 11.8 166.1 ± 94.9#	65.7 (38.9) -42.7 (20.4)	0.31 0.08

Tr (ms)	Left	ECC-CON-G CON-CON-G	42.3 ± 48.8 23.1 ± 29.8	110.8 ± 73.3 51.0 ± 44.6#	68.5 (161.9)^*^ 27.9 (120.9)	**0.48** 0.19

	Right	ECC-CON-G CON-CON-G	18.0 ± 17.6 44.8 ± 75.9	83.6 ± 59.5 49.7 ± 75.0	65.6 (364.6)^*^ 4.9 (11.0)	**0.55** 0.01

Td (ms)	Left	ECC-CON-G CON-CON-G	18.6 ± 1.2 17.3 ± 1.7	20.7 ± 1.1 20.4 ± 1.8	2.1 (11.5)^*^ 3.0 (17.6)^*^	**0.63** **0.70**

	Right	ECC-CON-G CON-CON-G	17.1 ± 1.9 17.9 ± 1.5	21.3 ± 1.1 19.8 ± 1.7#	4.3 (24.9)^*^ 1.9 (10.5)^*^	**0.76** **0.56**

Dm (mm)	Left	ECC-CON-G CON-CON-G	1.1 ± 0.9 0.9 ± 0.6	2.8 ± 0.7 2.5 ± 1.0	1.7 (155.9)^*^ 1.6 (182.2)^*^	**0.72** **0.73**

	Right	ECC-CON-G CON-CON-G	0.8 ± 0.8 0.8 ± 06	3.0 ± 1.1 2.0 ± 0.8#	2.1 (256.6)^***^ 1.1 (135.3)^*^	**0.75** **0.62**

Legend: M – mean; Sd – standard deviation; pre-test; initial measurement; Post-test – final measurement; Diff – difference between the initial and final measurements; ηp^2^ – partial eta square; Tc – contraction time; Ts – sustain time; Tr – relaxation time; Td – delay time; Dm – maximal displacement; ECC-CON-G – Eccentric plyometric intervention group; CONC-CON-G – Concentric plyometric intervention group;

* – statistically significant result, p < 0.05;

** – statistically significant result, p < 0.001.

**FIG. 3:**
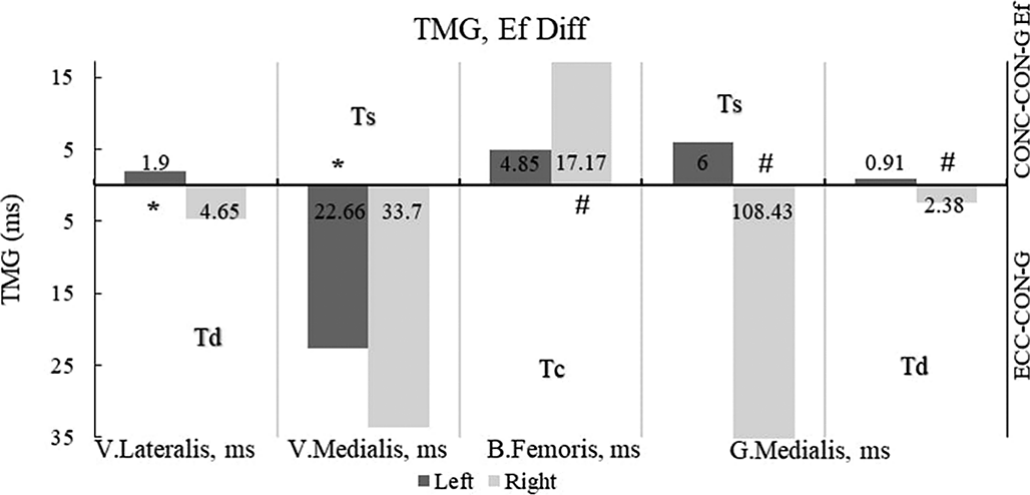
Differences in the effects of the plyometric programs on TMG parameters Legend: Td-delay time; Ts-sustain time; Tc-contraction time; * – statistically significant result of right leg set with p< .05; # – statistically significant result of left leg set with p< 0.05; ECC-CON-G – Eccentric plyometric intervention group; CONC-CON-G – Concentric plyometric intervention group. Note: Ef Diff were calculated by subtracting the results of the mean values ECCCON-G from CONC-CON-G. All results are presented as absolute values on both sides of the Y-axis: below the X-axis as the ECCCONC-G effect side, and above the x-axis as the CON-CONC-G effect side.

Figure 3 displays the effects on the left lower leg extensor muscle, VL, with a large effect for Td, and on VM for Ts. The remaining muscles did not show significant differences for any TMG parameters. Except for the right calf flexor muscle, BF for Ts, and the right foot extensor muscles, GM for Ts and Td, the other muscles in the right leg did not show any statistically significant results.

## DISCUSSION

To our knowledge, this is the first study to examine the effects of training programmes with TMG parameters in young female athletes, junior age competitors from various sports. Several longitudinal studies have used TMG analysis to examine one or eight muscles of the lower limbs in older athletes [8–13]. Another novelty of this study is the experimental design incorporating involuntary maximum ECC-CON and DL exercises, aimed at developing strength and speed rather than joint stability.

At the initial measurement, the ECC-CON-G and CONC-CON-G had similar anthropometric characteristics, strength, and speed. The only differences were observed in the left leg VL (Dm), and the right leg BF (Ts).

In most studies that examined the effects of different plyometric programmes on biomechanical parameters [2, 3, 36], strength parameters [19], and speed [37], the groups were the same at the initial measurement, which is also the case here.

### Post-test

The results showed significant differences at the final measurement between the two groups for the CMJ kinetic parameters EccV, ConcT, and Time, as well as in both speed test results at 10 m and 20 m. The following TMG results are significant for the left leg: lower leg extensors for VL (Td), VM (Tc), calf flexor BF (Dm), and GM foot extensor muscle (Ts and Tr). For the right leg, significant results were found for the calf flexor muscle BF (Tc) and foot extensor GM (Ts, Td, and Dm).

There are studies that examined the effects of experimental plyometric programmes with a control group, and a smaller number that examined different plyometric programmes. Several studies have obtained results similar to ours for CMJ parameters and speed. One study [36] showed differences at the final measurement in CMJ height between groups, similar to our findings [38].

The explanation for the results obtained in our study lies in the differences in the design of the plyometric programmes. ECC-CON and DL have a completely different effect on the neuromuscular system and the CNS, as they require an involuntary maximum effort from athletes, in contrast to CONC-CON or SJ exercises [18, 39]. Similarly, CONC-CON and SJ exercises involve a greater maximum voluntary contraction, but this is still limited compared to ECC-CON [39], partly due to the lower force produced when performing an SJ compared to a DL [1].

### Difference between pre- and post-test

Significant differences between the initial and final measurements were observed in the ECC-CON-G for CMJ in three parameters: Height, Rel F, and Rel I. In the CONC-CON-G, significant results were found for two parameters: Height and Rel I, as well as for the 10 m and 20 m sprint test results.

The success of the plyometric training method and the improvement in CMJ, ranging from 2 to 13 cm [37, 38, 40], or from 6% to 12% [2, 4, 22], was confirmed. For speed tests, the improvement at 10 m was 5% [22], and at 20 m, it ranged from 6 ms to 11 ms [4], or 5.7% [36]. The reason for the progress is in the plyometric way of training, which is made up of plyometric action, that is, the stretchshortening cycle (SSC). Specifically, in the third phase, known as the “coupling time”, the limit values of 15 ms refer to the knee joint [41]. This phase is the key to plyometric performance, because the shorter the cushioning phase, the more efficient and powerful the plyometric movement, i.e. the stored energy is used more efficiently in the transition from eccentric to concentric contraction [1, 27]. In this way, the neuromuscular system develops and adapts for the next stronger training, i.e. the jump, where greater stored elastic energy is used [1, 28]. Marković and Mikulić [42] summarized the effects of plyometric training (PT) as follows: (a) positive adaptation through increased neural drive to the agonist muscles; (b) enhancement of muscle activation patterns; (c) adjustments in the mechanical characteristics of the muscle-tendon complex of the plantar flexors; (d) changes in muscle size and structure; and (e) optimization of singlefibre kinetics, and improved TMG lateral symmetry [43]. Also, the best confirmations are the results of meta-analyses showing ES for CMJ height of 4 cm (ES = .84 [20] to ES = 1.24), [5] or with high intensity training 5 cm (ES = 1.22), [20], improved speed, sprints at 10 m (ES = 1.67), and over 10 m (ES = .92) [5].

Fourteen TMG parameters showed significant differences and effects in the muscles of the left leg in the ECC-CON-G, across all six measured muscles. For the right leg, twelve TMG parameters were significant across four muscles, excluding VM and SM. In the CONC-CON-G, six TMG parameters showed significant differences and effects in the muscles of the left leg. For the right leg, twelve TMG parameters showed significant effects across five measured muscles, excluding SM.

The scarcity of TMG studies that included younger athletes limited the possibility for comparison. We only found one study that examined the effects of a plyometric programme on athletes using TMG analysis is [11]. With a significant improvement in the CMJ jump, there was a notable decrease in Tc and muscle tone. The height of the CMJ jump increased significantly by 12.2%, while in VL, Tc decreased by 8.7%. A significant reduction of 26.7% was recorded in BF and 25.8% in GL, but no significant change was observed in GM. For Dm, there was a significant decrease in BF by 26.5%, in GM by 14.9%, and in GL by 31.5%, but no significant change was observed in VL. The results of the current study do not align with those of Tc (for VL, BF, GM, and partially align in GL) or Dm (for BF). While there is a statistically significant difference in the Tc results (for GM) and Dm results (for GL and GM), these showed a significant increase rather than a decrease. The reason for this mixed result may be that the subjects were men aged 20 to 40 who practised sports recreationally. The training consisted of only simple jumps without any variation in the exercises. Plyometric jumps had the goal of minimizing ground contact time. Also, the control group had its own activities that constituted different training, so it is not possible to state with certainty the intensity of its training or the types of jumps performed. Additionally, the difference lies in the fact that both of our plyometric programmes incorporated exercises that emphasized either CONC-CON or ECC-CON.

Another TMG analysis study, conducted with older recreational participants [12], found a significant improvement of 8.2% in the CMJ jump, with a notable reduction in Tc, ranging from 5.7% to 28.9% in GM and BF. However, no significant difference was observed in GL. A significant decrease of 9.6% for Tc in GM aligns with the current study, as well as the nonsignificant results for GL and VL. Partial alignment was observed for the reduced time in BF, at 5.7%. The smaller reductions in Dm compared to the current study are not consistent with the research findings. One of the reasons for these results is that the study involved older recreational athletes aged over 65, who underwent controlled plyometric training intensity to examine neuromuscular changes and TMG parameters. There is evidence suggesting that aging, along with muscle disuse, negatively affects the functioning of the neuromuscular system and reduces work capacity [44]. Additionally, aging is associated with the loss of muscle fibres and motor neurons, as well as atrophy of predominantly fast-twitch muscle fibres, leading to a decrease in overall muscle mass, slower Tc, and reduced muscle power [45]. Additionally, neither study [11, 12] specified when exactly TMG measurements were made, as the washout period after heating could potentially influence the results.

### Differences in effects between the two plyometric programmes

The plyometric programme significantly contributed to better results in the ECC-CON-G compared to the CONC-CON-G, for the kinetic parameters of CMJ, Height, and Time, speed at 20 m, as well as in the left leg VL (Td), VM (Ts), the right leg BF (Tc), and GM (Ts and Td).

The plyometric method makes different use of the stored forces in the muscles, tendons, and ligaments caused by the first main part of the training [27]. This is supported by the results of meta-analyses, which show that different types of exercises or jumps have varying effects. The SJ contributes to an increase of 3 cm (ES = 0.54), the depth jump adds 4 cm (ES = 0.66), CMJ contributes slightly less than 3 cm (ES = 0.41), while a combination of different jumps yields the best results [20].

Results consistent with the current study can be found in a study [38] which detected differences in the effects between the experimental groups, favouring the third exercise programme with high intensity in terms of jump height, kinetic strength parameters, and jump time.

There are studies that have examined the effects of exercises specifically with ECC-CON and CONC-CON [10, 19, 26], but few have used DL to develop strength and speed [23]. In the first group, after performing 475 DL from extreme heights of 2 m to 3.2 m over 12 weeks of training, significantly better results were achieved compared to the other exercise programme, which included 1,472 different plyometric jumps, SJ, and other resistance exercises. The results demonstrate significant effects on the development and increase of both eccentric and concentric strength. The muscle action during landing, lasting 28–61 ms, generates a force 20 times the body weight, which is stored in the neuromuscular complex and later transformed into functional force through further exercise, resulting in improved explosive power and speed [1, 23]. The contact time is too brief, and the forces are too immense for the neuromuscular system to modify the response. Any attempt to reduce the external force occurs by preparing the CNS before contact with the surface. The greater the force during landing, the more energy is absorbed by other parts of the skeletal muscles throughout the body [24].

The level of muscle activation during maximum voluntary contraction is lower in exercises with ECC-CON compared to CONC-CON. However, when performing a greater number of consecutive repetitions, eccentric exercises result in less muscle fatigue compared to concentric exercises with the same principle [39, 46]. Paradoxically, the maximum voluntary force during ECC-CON is greater compared to CONC-CON [46]. ECC-CON, as a stimulus, is essential for inducing muscle hypertrophy [46], particularly in type II muscle fibres, with up to 10 times more hypertrophy occurring compared to concentric training [26].

Involuntary maximum contractions can only be performed during eccentric exercises, such as DL, which directly leads to advantages over exercises with CONC-CON [39]. Another benefit of landing is that, in the first 50% of the ECC-CON phase, most of the negative work of the muscles and joints occurs. As landing height and impact speed increase, the time required for the work decreases, accelerating the action of the neuromuscular system that absorbs kinetic energy [1, 39, 27]. With greater muscle tone and stored force in the lower extremities, it becomes possible to achieve higher running speeds and activate the contractile properties of muscle fibres for a longer duration [47].

The plyometric training method, involving various double-leg jumps, enhances strength and speed development while equally improving neuromuscular responses in the muscles of both legs [1, 28, 48]. The presence of differences in strength and neuromuscular response between the dominant and non-dominant legs is a normal phenomenon in athletes. A strength difference of up to 15% indicates a typical inter-limb asymmetry [49]. Plyometric training has a positive effect on improving inter-limb symmetry in athletes, particularly in younger athletes who do not experience fatigue from overusing one limb due to the technical demands of their sport [43, 48]. Although our study shows improvements in TMG parameters for both the left and right legs, we do not have data on the dominant and non-dominant legs. However, we can conclude that there is similar progress in both legs for most of the measured muscles and TMG parameters.

### Limitations and strength

The first limitation of the study was the small number of participants, and the second limitation was the lack of data on the participants’ dominant jumping leg and arm. Depending on the hand used for actions such as the two-step shot, spike, or serve, the dominant rebound leg can be determined based on the specifics of performing these technical elements [50]. Since most participants have a dominant left leg or are right-handed, it can be hypothesized that the programme significantly improves the motor response and muscle tone of the left leg, which acts as the rebound leg in the ECC-CON-G. Additionally, the pre-existing levels of explosive strength and speed in the test participants’ muscles are further enhanced, leading to greater hypertrophy and activation of type II fast-twitch muscle fibres [19, 26, 46].

## CONCLUSIONS

Our 6-week study, with 2 high-intensity training sessions per week, confirms previous findings on the effectiveness of the plyometric training method and adds to the limited research on younger athletes, particularly the female population. This study offers a new approach to using involuntary maximal contractions through pseudo-plyometric exercises, specifically landings without jumping from relatively lower-risk heights compared to previous research. This method supports the development of speed, strength, and the capacitive ability of the muscles in the lower extremities. The increased storage and charge in the neuromuscular system from DL create a state that allows for faster and more effective progress compared to exercises based solely on CONC-CON, such as the SJ. It can be concluded that a plyometric programme based on exercises with ECC-CON produced significantly better results compared to a programme based on exercises with CONC-CON, particularly in terms of strength, speed, and TMG parameters in young female athletes. While it is not yet certain whether DL from greater heights, combined with plyometric exercises, contributed to better results due to neural adaptation and greater force scaling in the neuromuscular system, or because of the potential development of fast-twitch muscle fibres (type IIa or IIb), which are most activated during eccentric training, we recommend that future studies explore this question further.
